# Polymerase chain reaction - surface-enhanced Raman spectroscopy (PCR-SERS) method for gene methylation level detection in plasma

**DOI:** 10.7150/thno.30204

**Published:** 2020-01-01

**Authors:** Xiaozhou Li, Tianyue Yang, Caesar Siqi Li, Youtao Song, Deli Wang, Lili Jin, Hong Lou, Wei Li

**Affiliations:** 1 School of Science, Shenyang Ligong University, Shenyang 110159, China; 2 College of Environmental Sciences, Liaoning University, Shenyang 110036, China; 3 College of Medicine, Northeast Ohio Medical University, Rootstown 44272, USA; 4 School of Life Science, Liaoning University, Shenyang 110036, China; 5 School of Electronic Science and Engineering, University of Electronic Science and Technology of China, Chengdu 611731, China

**Keywords:** PCR, methylation level, plasma, surface-enhanced Raman spectroscopy, SERS

## Abstract

Gene promoter hypermethylation is a vital step in tumorigenesis. This paper set out to explore the use of polymerase chain reaction - surface-enhanced Raman spectroscopy (PCR-SERS) for the detection of gene methylation levels, with a focus on cancer diagnosis.

**Methods:** PCR with methylation independent primers were used on DNA samples to amplify target genes regardless of their methylation states. SERS was used on the obtained PCR products to generate spectra that contained peak changes belonging to CG and AT base pairs. Multiple linear regression (MLR) was then used to deconvolute the SERS spectra so that the CG/AT ratios of the sample could be obtained. These MLR results were used to calculate methylation levels of the target genes. For protocol verification, three sets of seven reference DNA solutions with known methylation levels (0%, 1%, 5%, 25%, 50%, 75%, and 100%) were analysed. Clinically, blood plasma samples were taken from 48 non-small-cell lung cancer (NSCLC) patients and 51 healthy controls. The methylation levels of the genes p16, MGMT, and RASSF1 were determined for each patient using this method.

**Results:** Verification experiment on the mixtures with known methylation levels resulted in an error of less than 6% from the actual levels. When applied to our clinical samples, the frequency of methylation in at least one of the three target genes among the NSCLC patients was 87.5%, but this percentage decreased to 11.8% for the control group. The methylation levels of p16 were found to be significantly higher in NSCLC patients with more pack-years smoked (p=0.04), later cancer stages (p=0.03), and cancer types of squamous cell and large cell versus adenocarcinoma (p=0.03). Prediction accuracy of 88% was achieved from classification and regression trees (CART) based on methylation levels and states, respectively.

**Conclusion:** This research showed that the PCR-SERS protocol could quantitatively measure the methylation levels of genes in plasma. The methylation levels of the genes p16, MGMT, and RASSF1 were higher in NSCLC patients than in controls.

## Introduction

DNA methylation refers to the addition of a methyl group at the 5th position of the cytosine ring, which often causes gene silencing and noncoding 1. It is one of two mainstays of human epigenetics and plays a significant role in the development of cancers 2, 3. Cancer-related genes include tumour suppressors, DNA repair genes, metastasis genes, and others which all may be affected by DNA methylation 3. Consequently, DNA methylation has been used as a biomarker for cancer diagnosis, prognosis, and pharmacoepigenetics 4.

Several methods have been proposed to detect methylation levels. Those methods can be categorised as bisulfite-based, restriction enzyme-based, and affinity-based 5. Among those, bisulfite-based methods are the most popular and well-established approach. This kind of approach utilises bisulfite conversion to change unmethylated cytosine (C) to uracil (U) while leaving methylated C unchanged. However, methylation-specific polymerase chain reaction (MSP) 6, 7 and methylation-sensitive denaturing high-performance liquid chromatography (MS-DHPLC) 8 provide only qualitative detection of DNA methylation. While, methylation-sensitive single nucleotide primer extension (MS-SnuPE) 9, 10, combined bisulfite restriction analysis (COBRA) 11, 12, and MethyLight 13, 14 can only quantify known CpG sites. These methods achieved the goal of separating molecules and measuring methylation levels by exploiting differences in molecular charge, size, structure, and configuration. However, they suffer from disadvantages such as high cost, low sensitivity, and complexity in selecting DNA amplification reaction conditions. For example, bisulfite-sequencing 15, 16 has high equipment requirements, and the detection process usually takes several days. And the method of COBRA and bisulfite-sequencing require the use of enzymes which increase expense and reduce the practicality for routine testing. The aim of this article was to explore the introduction of surface-enhanced Raman spectroscopy (SERS) - an optical spectroscopy method - for the detection of methylation levels in combination with PCR.

SERS is a type of Raman spectroscopy that operates by placing samples near rough noble metal surfaces called SERS substrates. Raman scattering was first observed by Raman and Krishnan in 1928 17. It can provide “fingerprint” patterns of target molecules due to inelastic vibration origin. Since water is Raman-inactive, aqueous samples can be examined using Raman techniques without any pretreatment. This feature makes the Raman technique suitable for bio-fluid detection 18. However, sample degradation and fluorescence disturbance are the main challenges in detection using Raman scattering. While SERS can avoid the problems of normal Raman mentioned above, and can enhance Raman signals up to an order of 14 magnitudes because of the plasmonics effect on the surface of SERS substrates 19. Moreover, SERS has narrower peaks which can reduce peak overlap, and is straightforward to be incorporated with other biological techniques 20.

Due to the stated benefits, SERS has been widely used in the fields of tissue imaging 21, biomolecule monitoring 22, and liquid biopsy 23. In the realm of DNA and gene detection, SERS has successfully detected nucleic acids ranging from mononucleotides 24, oligomers 25, 26, and entire genes 27-29. SERS for DNA detection uses either direct (without Raman tags) or indirect (with Raman tags), and dependent (combined with other biological techniques) or independent methods 30, 31. In the previous study, SERS has been successfully used for the detection of blood gene mutation by using mutation-specific PCR and Raman tags 32-35. Direct SERS methods which can provide DNA modification-specific spectra are typically adopted for the detection of methylation of short DNA sequences 25, 36-38. A target amplification method is usually used before SERS to detect methylation levels of certain genes. For instance, Wang et al. measured single base methylation changes of as low as 10% employing SERS after bisulfite PCR and ligase chain reaction (LCR) 39. Single base methylation was measured by comparing the intensities of the methylated-specific LCR reaction against that of the unmethylated-specific LCR reaction.

This article introduces a PCR-SERS method to measure methylation levels of target genes. As cytosine undergoes deamination to uracil, methylation levels may be calculated by merely measuring the CG percentages of the PCR product. CG percentages were calculated using multiple linear regressions (MLR), with SERS of CG and AT being used as reference spectra. This PCR-SERS protocol was first verified on 21 reference DNA mixtures containing known methylation levels. For each of the three lung cancer-related target genes (p16, MGMT, and RASSF1), seven mixtures were created by varying the amount of the methylated to the unmethylated gene of interest (0%, 1%, 5%, 25%, 50%, 75%, and 100%). After this verification step, the PCR-SERS protocol was then applied to detect the methylation levels of p16, MGMT, and RASSF1 in actual plasma samples taken from 48 non-small-cell lung cancer (NSCLC) patients and 51 controls. Finally, the correlation between the methylation levels of the three genes in each sample and clinical characteristics of each patient were analysed with Fisher's exact test. The diagnostic ability of the methylation levels of the three genes was evaluated using receiver operating characteristic (ROC) analysis and classification and regression trees (CART).

## Materials and methods

### CG and AT sequences

Since the SERS spectra of double-strand CG (dsCG) and AT (dsAT) will be used as the reference spectra in the MLR analysis, the SERS of dsCG and dsAT were first measured. Oligonucleotides of dsCG and dsAT were purchased from Sangon Biotech (Shanghai, China). The preparation of the oligomer sequences (Table 1) was performed in accordance with the method described by Guerrini, et al 36. Solutions of dsCG and dsAT were prepared by heating complementary strands to a temperature of 95 °C for 10 min. The concentration of the final dsCG and dsAT was 10^-5^ M, and the obtained double-strands were stored at -20 °C.

### Reference DNA solutions

For each gene of interest (p16, MGMT, and RASSF1), seven DNA mixtures with increasing methylation levels of 0%, 1%, 5%, 25%, 50%, 75%, and 100% were prepared. Methylated genes were prepared using CpG Methyltransferase (M.SssI) according to the vendor's instructions. Standard DNA mixtures were created by mixing the totally methylated DNA gene solution with the unmethylated solution in the ratios of 0%, 1%, 5%, 25%, 50%, 75%, and 100%.

### Plasma DNA samples

Plasma DNA was extracted from the blood plasma of 48 NSCLC patients and 51 healthy controls with informed consent from Shengjing Hospital of China Medical University (Shenyang, China). Medical Research Ethics Committee of Shengjing Hospital approved all experimental protocols. Table 2 shows the clinical features of the 99 volunteers. For each patient, a total of 3 mL of peripheral blood was collected between 7:00 and 8:00 a.m. after a 12 h overnight fast. Collected blood was mixed with EDTA anticoagulant and was centrifuged at 5,000 rotations/min for 10 min at 4 °C to remove blood cells. Plasma was collected in a 1.5 mL Eppendorf tube and stored at -80 °C. Plasma DNA was extracted with a QIAamp DNA Blood Mini Kit (Qiagen, Hilden, Germany). The obtained DNA solution was stored at -20 °C.

### PCR

A PCR process was conducted before SERS. Prior to PCR, sodium bisulfite treatment was first conducted on all analyte DNA solutions: all three by seven sets of reference DNA mixtures with methylation levels of 0%, 1%, 5%, 25%, 50%, 75% and 100%, and all of the plasma-extracted DNA samples. PCR was then conducted on the bisulfite-treated DNA solutions using methylation-independent primers to amplify the three target genes separately without needing to consider the methylation states within the target gene slices (primer sequences are listed in Table 1). Each patient's plasma sample produced three DNA samples, one for each target gene. Based on the CpG points of the PCR products (Table 3), methylation levels from 0% to 100% had their corresponding CG percentages calculated as 54.84-62.37%, 34.69-46.94%, and 37.50-46.32% for p16, MGMT, and RASSF1, respectively. These subsequent changes in base pair percentages could be detected using SERS. Final PCR products were then purified using TIANGEN PCR purification kits (Tiangen, Beijing, China). Agarose gel electrophoresis indicated only one band of the expected size. All samples were run in triplicate for each assay.

### SERS

SERS spectra were recorded on an inVia Raman micro-spectrometer system (Renishaw, Great Britain) equipped with a He-Ne laser (λ=632 nm, beam diameter=1.5 μm), a RenCam CCD detector (400 × 575 pixels), and a Leica microscope with a 50× objective lens (NA=0.75). Laser power was set as 4 mW. The exposure time of the CCD was 10 s, and all the SERS spectra were evaluated in backscattering geometry by finding the average of three measurements. Smoothing, baseline correction, and normalization were applied to all spectra.

Spermine coated Ag colloids were made in accordance with the published method 40. Briefly, 10 mL AgNO_3_ (1 mM) and 5 µL spermine hydrochloride (0.1 M) were mixed under vigorous stirring, then 25 µL of NaBH_4_ (0.1 M) was added to the solution and stirred for 20 min. Spermine functioned as an aggregating and DNA backbone neutralising agent in this experiment 41. Figure S1 shows the TEM image of the silver nanoparticles and the corresponding size distribution plot. Samples were prepared by mixing 60 µL of the Ag nanoparticles and 10 µL of the PCR products and were sucked into capillaries (i-Quip, USA) for spectral collection.

### Statistical analysis

All data analyses were carried out using the open-source R programming language (https://www.r-project.org/). MLR was used on the SERS spectra of PCR products to decompose them into spectra of dsCG and dsAT 42. In the MLR, the SERS spectra obtained from PCR-SERS was treated as target spectra (dependent variables), and the SERS spectra of dsCG and dsAT were used as reference spectra (independent variables). Whole spectra were used as input for MLR. Since the spectra are baseline corrected, polynomial background per Lutz and Vo-Dinh 43, 44 were not considered in this paper. ROC analysis was used for the calculation of specificity, sensitivity, and accuracy of the methylation levels of each of the three genes for cancer diagnosis. ROC was performed using the R package pROC 45. Optimal cut-offs were determined by the threshold that maximises the distance calculated by Youden's J statistic provided by the “coords” function (“best.methods” argument) of pROC package 46. CART were used on the obtained methylation levels and methylation states of all three genes to determine their diagnostic ability. In the process of forming the decision tree, CART splits data recursively on a dichotomous basis until specific criteria are met 47. This feature makes it suitable for analysing the relationship between methylation levels of genes and clinical features. Fisher's exact test was used to determine the correlation between methylation levels of the three genes (p16, MGMT, and RASSF1) and the clinical characteristics of the corresponding NSCLC patients.

## Results

### PCR-SERS method

**Figure 1:**
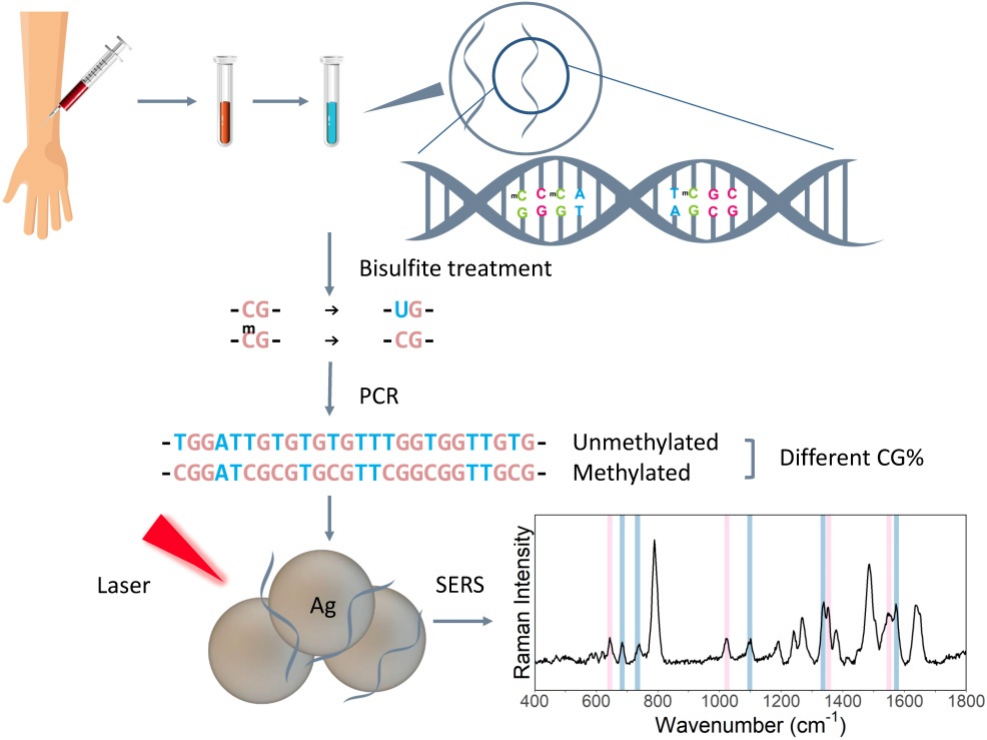
Schematic illustration of PCR-SERS method targeting gene methylation levels.

Schematic figure of the PCR-SERS method is presented in Figure 1. In summary, bisulfite treatment was first utilised on the DNA solution to convert unmethylated cytosine to uracil, then PCR was used to amplify the target genes. After that, the SERS of the PCR products was taken to detect the changes in base pair percentages. Finally, MLR was used to decompose the spectra into spectra of dsCG and dsAT. Thus, methylation levels could be calculated from these CG/AT ratios.

**Figure 2:**
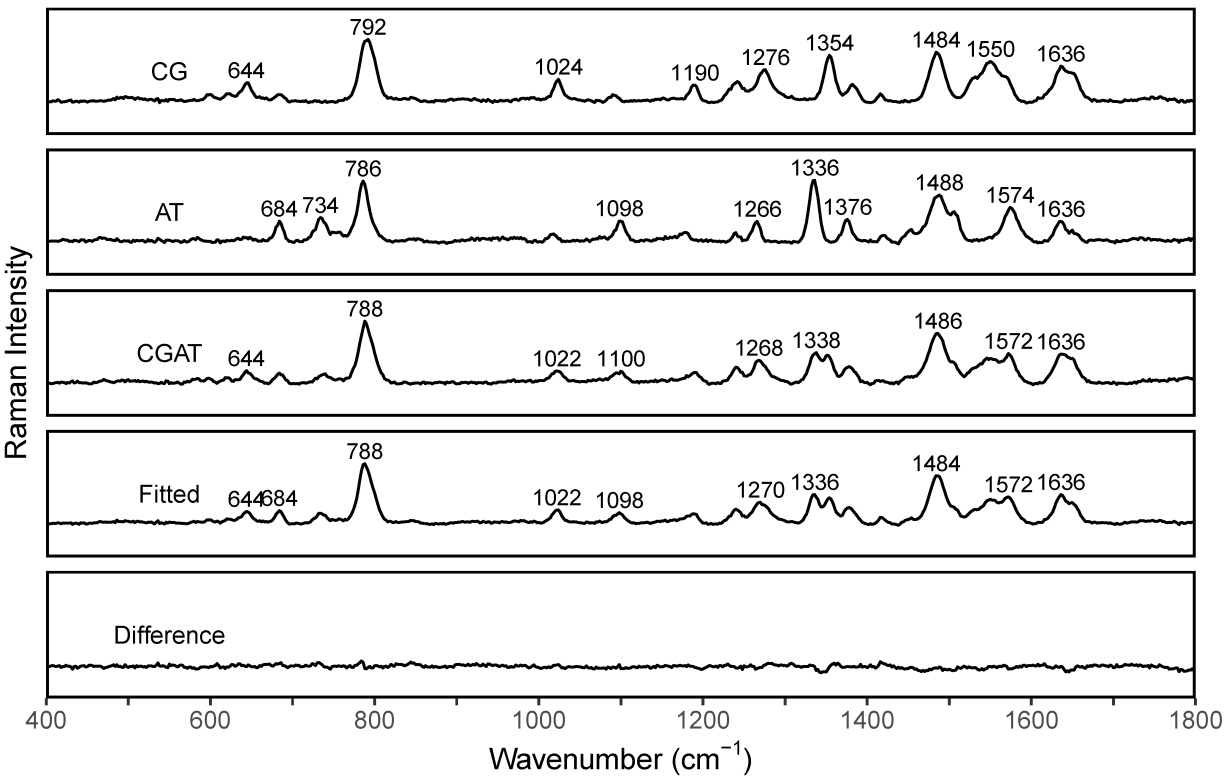
SERS spectra of dsCG (CG), dsAT (AT), dsCG/dsAT (CGAT), fitted spectra (Fitted), and difference spectra (Difference=Fitted-CGAT).

The differences in peak profiles in the spectra of dsCG and dsAT are the basis for the suggested PCR-SERS method. We measured SERS spectra of dsCG, dsAT, and dsCG+dsAT (with the ratio of 1:1) separately to see their quantification as reference spectra (Figure 2). The resulted spectra of dsCG and dsAT shared nine major peaks located at about 788, 1024, 1098, 1188, 1240, 1270, 1378, 1484, and 1636 cm^-1^. The four peaks at 644, 1024, 1354, and 1550 cm^-1^ belong to dsCG only, while the five peaks at 684, 734, 1098, 1336, and 1575 cm^-1^ belonged to dsAT only. These unique differences in peak profiles make the MLR analysis possible. MLR was used to deconvolute the SERS spectra of dsCG+dsAT using spectra of dsCG and dsAT as references. The fitted SERS of dsCG+dsAT showed good concordance with the original spectra (as shown in Fitted and Difference spectra in Figure 2).

### Performance of PCR-SERS

**Figure 3:**
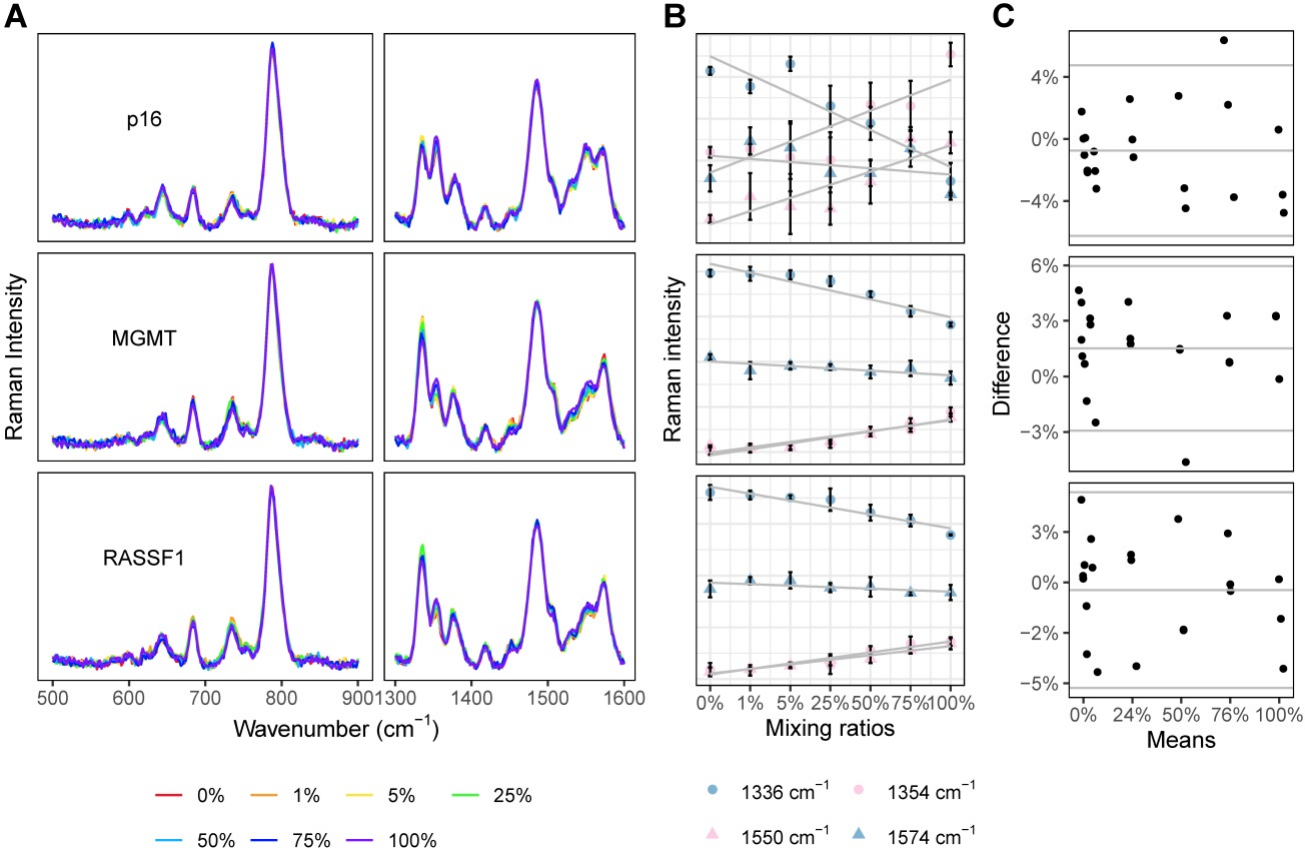
PCR-SERS were tested on standard DNA solutions to calculate CG percentages and subsequent methylation levels. **(A)** SERS spectra of DNA mixtures of three genes (p16, MGMT, and RASSF1) with increasing methylation levels (0%, 1%, 5%, 25%, 50%, 75%, and 100%). **(B**) Peak height changes of the four feature peaks at 1354 and 1550 cm^-1^ (belonged to dsCG) and 1336 and 1574 cm^-1^ (belonged to dsAT). **(C)** Bland-Altman plots comparing calculated methylation levels and the actual ones. For each subplot, the x-axis represents the mean of the calculated and actual values, and the y-axis represents the difference between the two values.

For each gene of interest (p16, MGMT, and RASSF1), seven prepared solutions with variable methylated gene content (0%, 1%, 5%, 25%, 50%, 75%, and 100%) were analyzed with the PCR-SERS method. The SERS profiles of these standard solutions were grossly similar (Figure 3A). The four unique peaks of dsCG (1354 and 1550 cm^-1^) and dsAT (1336 and 1574 cm^-1^) showed consistent and expected changes with increases in methylation levels (Figure 3B). For all three genes, the dsCG peaks at 1354 and 1550 cm^-1^ (red) increased, while dsAT peaks at 1336 and 1574 cm^-1^ (blue) decreased with increasing methylation.

The composing percentages of CG and AT as obtained from the SERS spectra (CGAT) were then analysed using MLR. Whole spectra were used as input for MLR. The output variables of the MLR were two coefficients for each reference SERS spectrum (spectra of dsCG and dsAT). These two coefficients represented the composing percentages of dsCG and dsAT, respectively. The resultant MLR coefficients, calculated methylation levels and the differences between the calculated and the actual methylation levels are listed in Table S1. Bland-Altman plots showed that all methylation estimates fell within 0.06 (6%) deviation from the actual values (horizontal parallel lines in Figure 3C), indicating good concordance across all of our reference DNA solutions. The precision of our estimations did not change with the levels of methylation or with different gene types. To verify that the change in the SERS spectra resulted only from changes of base pairs, we compared the peak heights obtained from raw spectra with those from processed spectra across our mixtures (0%, 1%, 5%, 25%, 50%, 75%, and 100%). Results showed that the peak heights and trends in the bands at 1336, 1354, 1550, and 1574 cm^-1^ were similar between processed and raw spectra. The averaged peak heights of CG and AT from our raw and processed spectra were compared using a paired T-test. For each gene, p-values exceeded 0.05 (p=0.07, 0.82, and 0.86), which indicated that there was no significant difference between the raw and processed spectra (Figure S2).

### Clinical application

**Figure 4:**
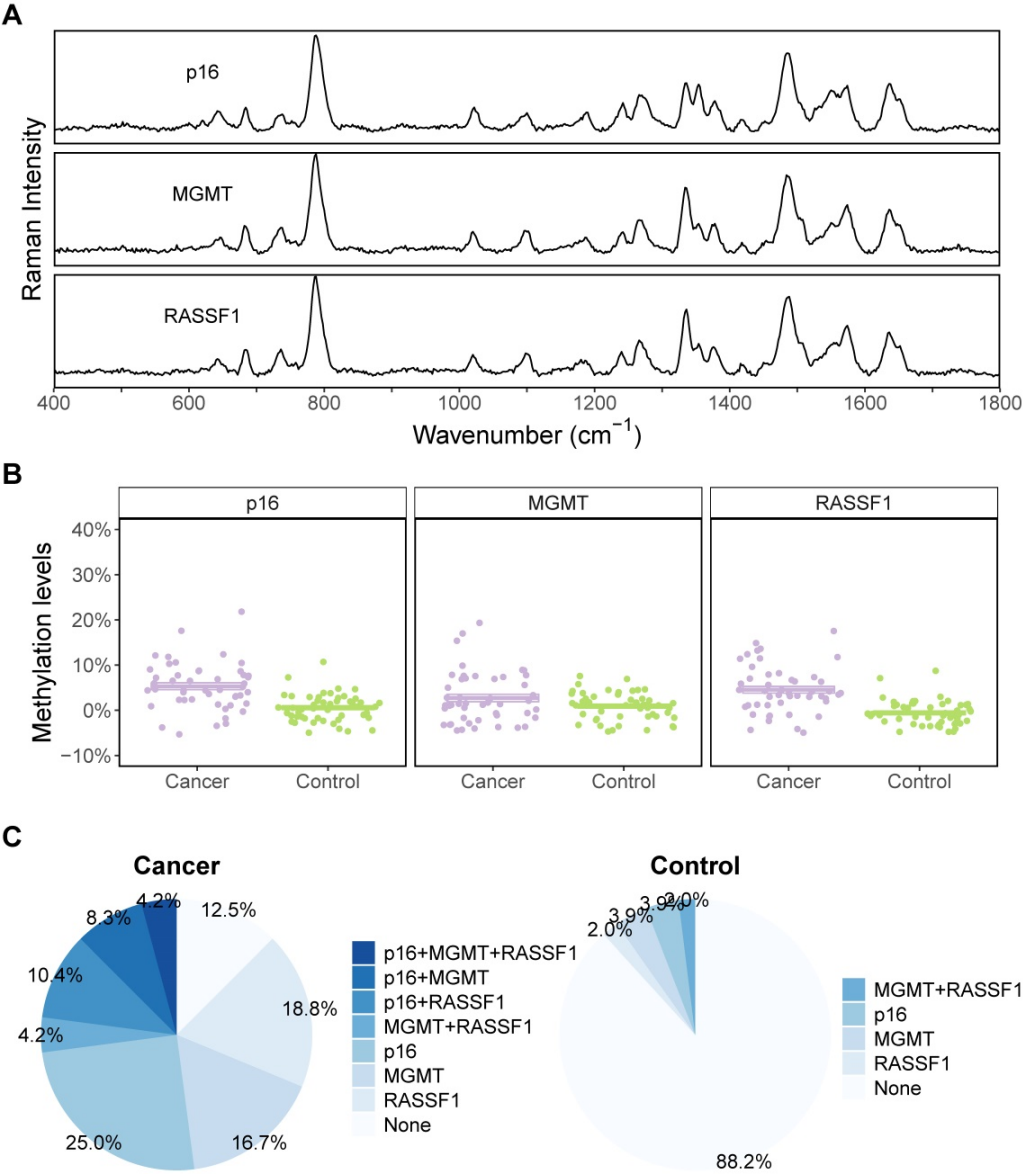
NSCLC cancer and control group showed different methylation profiles. **(A)** Representative SERS spectra of p16, MGMT, and RASSF1 got by PCR-SERS method. **(B)** Methylation level differences between cancer and control groups for the three genes of p16, MGMT, and RASSF1. **(C)** Pie plots illustrating methylation percentages of the three genes for cancer and control groups, respectively.

Plasma taken from 48 NSCLC patients and 51 controls were tested using the described protocol to evaluate the performance of PCR-SERS in real plasma samples. SERS spectra of plasma-extracted DNA were similar to those of the standard DNA mixtures (Figure 4A). The methylation levels of the samples of the three genes were obtained using MLR. For all analysed genes (p16, MGMT, and RASSF1), the methylation levels were found to be 5% higher in the cancer group compared to the control group (Figure 4B). In all three genes, 12.5% of the cancer group samples and 88.2% of the control group samples lacked methylation (Figure 4C). For the cancer group, 4.2% of the samples contained methylation in all three genes. However, the majority of samples only contained methylation in one gene. For the cancer group, the percentages are 25% for p16, 16.7% for MGMT, and 18.8% for RASSF1. While for the control group, those percentages were 3.9%, 3.9%, and 2% for p16, MGMT, and RASSF1, respectively.

### ROC and CART

The diagnostic ability of the methylation levels of the three genes (p16, MGMT, and RASSF1) was evaluated by ROC and CART analysis. First, the diagnostic ability of each single gene was evaluated by ROC analysis using the Youden index to determine the optimal cut-offs. ROC results showed that the diagnostic performance of any of the three genes was similar (Table 4). For individual genes, the specificity ranged from 33% to 69%, the sensitivity ranged from 92% to 94%, and the accuracy ranged from 65% to 80%. Sensitivity values were much higher than the specificity values. Low specificity and high sensitivity values indicated that the methylation levels of a single gene performed well for ruling out disease but may only positively identify cancer patients at the cost of high false positives.

**Figure 5:**
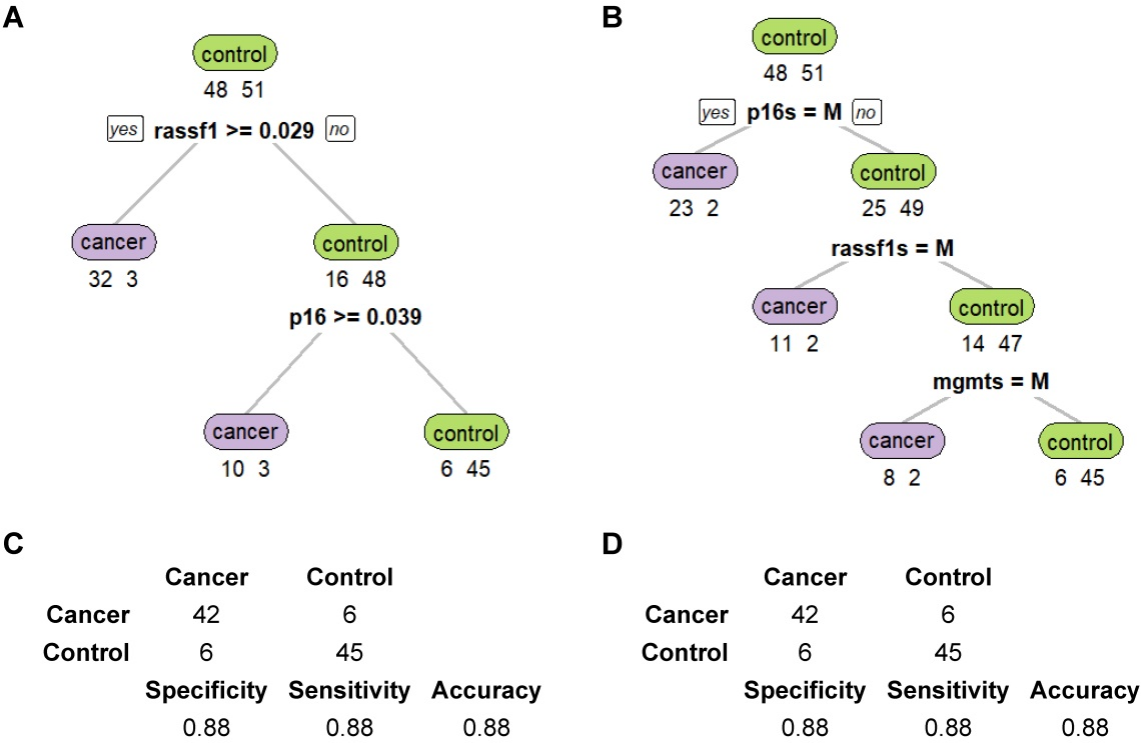
CART performances are the same for methylation levels and states in NSCLC cancer discrimination. **(A-B)** Decision trees of CART based on methylation levels and states. **(C-D)** Prediction results of CART based on methylation levels and states.

CART was then used to evaluate the diagnostic performance when methylation status of all three genes was combined. Both the methylation levels and the methylation states (whether methylation existed in the target genes) were evaluated for comparison. For methylation states, the genes were defined as methylated when methylation levels were at least 5% and were classified as unmethylated when methylation levels were lower than 5% (including negative values). Figure 5 depicts the structure of the two decision trees generated by CART. CART analysis split the samples to “Cancer” and “Control” groups stepwise based on the predictors of each tree level. The decision tree of methylation levels (Figure 5A) has two layers, while that of methylation states has three (Figure 5B). Figure 5C and D represent the diagnostic ability of each of the two CART trees. In general, CART performed better than ROC, and the results of methylation levels and methylation states in CART were the same. The specificity, sensitivity, and accuracy were all 88% for both methylation levels and methylation states.

**Figure 6:**
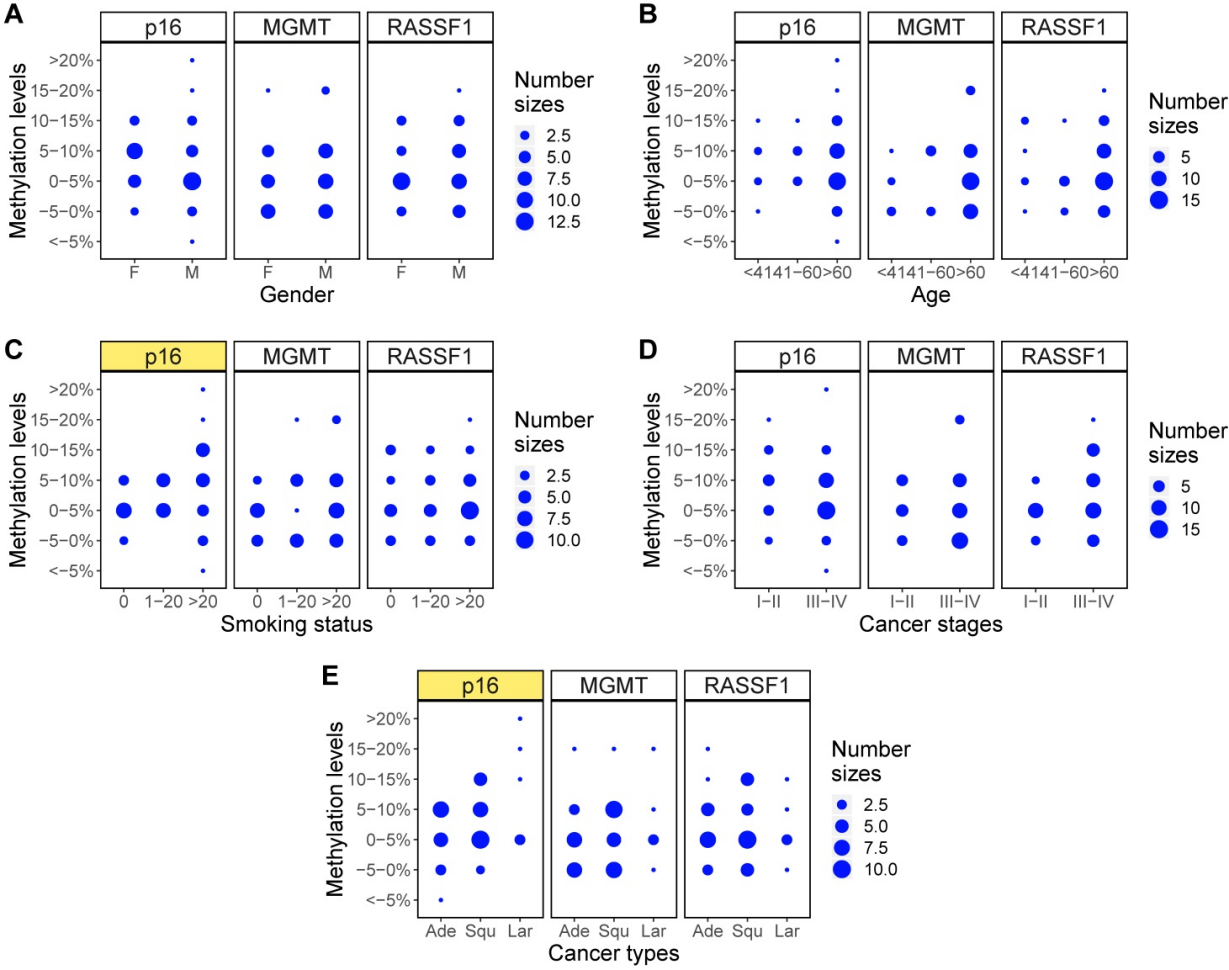
Methylation level changes for different clinical characteristics. **(A-E)** Balloon plots for methylation level distributions of p16, MGMT, RASSF1 for five clinical characteristics of gender, age, smoking status, cancer stages, and cancer types. Among those, methylation levels of p16 in different smoking status **(C)** and in different cancer types **(E)** showed significant differences (marked as yellow).

**Figure 7:**
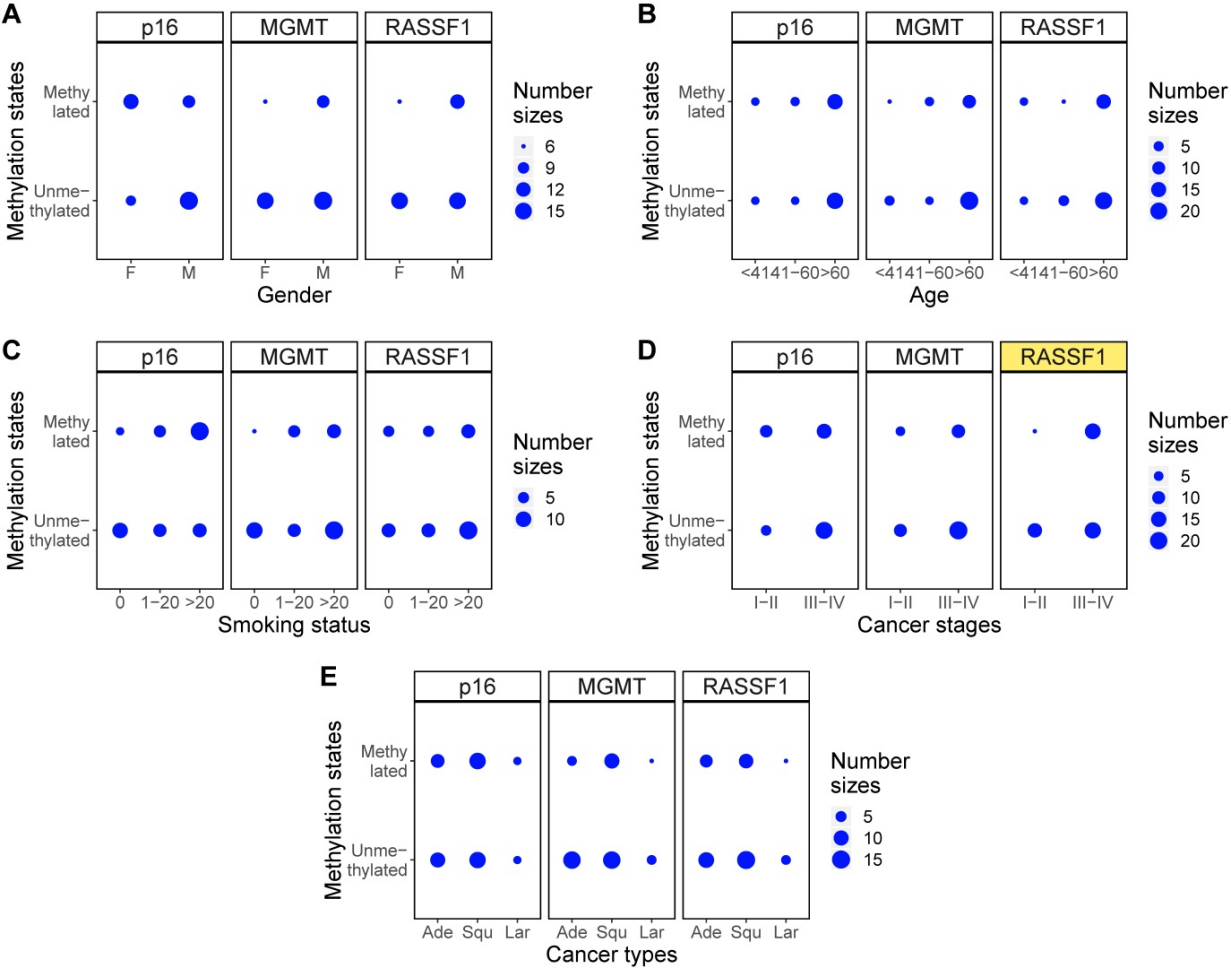
Methylation states changes for different clinical characteristics. **(A-E)** Balloon plots for methylation state distributions of p16, MGMT, RASSF1 for five clinical characteristics of gender, age, smoking status, cancer stages, and cancer types. Among those, methylation state of RASSF1 in different cancer stages **(D)** showed significant differences (marked as yellow).

Finally, methylation levels of the three genes were examined with Fisher's exact test to identify the relationship between gene methylation levels and clinical characteristics of the NSCLC patients. A comparison of the p values across each clinical feature showed that only the methylation levels of p16 across different smoking status (p=0.04), methylation states of RASSF1 across different cancer stages (p=0.03), and methylation levels of p16 across different cancer types (p=0.03) showed statistically significant differences (Table 5). Balloon plots show the visual differences in methylation levels (Figure 6) and methylation states (Figure 7). The subplots marked by yellow are the genes in which clinical features showed significant correlations with methylation. Specifically, methylation levels of p16 were higher for more pack-years smoked and were also higher in large cell and squamous versus adenocarcinoma. Methylation states of RASSF1 in advanced stages of cancer (stages III-IV) were higher than in early stages (stages I-II).

## Discussion

In this study, we demonstrated a PCR-SERS protocol that successfully detected methylation levels of certain genes in plasma samples. In this process, bisulfite processing was first used to convert unmethylated cytosine to uracil while leaving methylated cytosine unchanged, and then the low concentrations of target genes were amplified using PCR. The problem of DNA methylation detection became the problem of detecting CG percentage, which was solved with subsequent SERS analysis on the PCR products. As the peaks in these SERS spectra were contributed to by CG and AT base pairs, the SERS spectra were deconvoluted using MLR to get the composing percentages of dsCG and dsAT. Finally, based on the CG/AT ratios obtained from MLR, methylation levels were calculated. This PCR-SERS method was then verified by detecting the methylation levels of standard solutions with different methylation levels (0%, 1%, 5%, 25%, 50%, 75%, and 100%) of the three genes p16, MGMT, and RASSF1. A prediction error of less than 6% showed the high accuracy of this method. Subsequently, the PCR-SERS method was applied in clinical plasma samples taken from 48 NSCLC patients and 51 healthy controls. Peak assignments of reference DNA solution are listed in Table 6. The peaks were mainly contributed to by ring breathing vibration, backbone vibration, and in-plane vibrations of base residues. Raman spectroscopy will provide the information of secondary configuration of target molecules. Thus the adsorption situation between target molecules and SERS substrate will greatly influence the Raman shifts of SERS. For example, the drying process for dried samples and adsorption conformation for aqueous samples will all influence the adsorption conformation and then will change the Raman peak positions 48. It has been reported that the 5th atom in the pyrimidine ring of cytosine has been found to have four modification form: 5-methylcytosine (5-mC), 5-hydroxymethylcytosine (5hmC), 5-formylcytosine (5fC), and 5-carboxylcytosine (5caC) 49. Among those, 5-mC can be converted to 5hmC, and 5hmC can be further oxidized to 5fC and 5caC by ten-eleven translocation (TET) family of proteins. This means that the methylation states of genes are dynamic. While in the bisulfite treatment which is usually used before the methylation detection, both 5-mC and 5hmC will not change to uracil. Thus, in the bisulfite-based methylation detection such as in this paper, the methylations of genes were contributed by both 5mC and 5hmC. Further discrimination of the two types of methylated cytosines can be achieved by an additional treatment that will only affect one of the two forms of methylated cytosines 50.

Fisher's exact test revealed that p16 methylation levels correlated significantly with smoking status and the NSCLC cancer type in our patients. This result agrees with the report that higher p16 methylation levels were found in NSCLC patients with higher pack-years smoked 51-54. For different cancer types, the ranking of methylation levels at p16 from high to low was: large cell, squamous, and adenocarcinoma. This result is consistent with the findings described in references 53, 51, 54 which found that p16 methylation was more frequently observed in squamous carcinomas than in adenocarcinomas. The methylation of MGMT did not show correlation with any clinical features in this experiment. This finding differs from the results presented in the reference 55 which showed that MGMT methylation occurred more frequently in adenocarcinoma and increased significantly with tumour progression. In terms of the RASSF1 gene, our results indicated that methylation states displayed a significant increase that correlated with the progression of cancer stages, which is consistent with previous reports 56, 57. Consistent with the literature 57, we noted no statistical difference in RASSF1 methylation levels between different NSCLC cancer types. Diagnostically, the results of CART showed that the prediction accuracy of methylation levels and methylation states were the same (88%), which indicated that the use of methylation levels did not provide more algorithmically useful cancer information than methylation states. This may be because the decision of gene methylation states in our paper is based on gene methylation levels (≥5% as methylated, and <5% as unmethylated).

With respect to DNA detection using SERS, target amplification methods that examine methylated points are generally used. Previous studies have used LCR 39 and single-base extension reaction 58 respectively to amplify methylated and unmethylated genes, and predicted the methylation states of the gene points successfully with the help of Raman tags or intensities of Raman peaks. In comparison, through the use of PCR, the detection targets of PCR-SERS in this paper are the percentages of the methylated base pairs in the gene sequences. Table S2 compares the details of the PCR-SERS and other conventional methylation detection methods. Methylation detection was mainly achieved in two ways - enzymes (e.g., methylation-sensitive restriction endonucleases) and chemical reactions (e.g., bisulfite, hydrazine, and permanganate) 59, only methods utilising bisulfite pretreatment were compared in this paper. Unlike conventional methods, SERS can provide quantitative information about the methylation levels between primers rather than at predetermined CpG sites. No post-PCR process such as gel electrophoresis was needed. As an added benefit, as the detection method, the whole process of SERS can be finished within minutes. The detection limit of PCR-SERS (about 6%) is higher than that of MS-HRM (0.1%) which is also a quantitative methylation level detection method 60. The instability of SERS substrates and the complex interactions between the analyte and SERS substrate may be the contributing factors. SERS substrate with high reproducibility and uniformity is an area of improvement in future research.

Though PCR-SERS in this paper can measure methylation levels (percentages) of a target gene sequence, it does not specify the methylation positions. Another drawback of this method is that it cannot perform multiplex gene detection. Further investigation into other target amplification methods used in combination with SERS is recommended. In addition, this assay at present may not be used in clinical diagnosis as the SERS spectra heavily depend on the uniformity of SERS substrate. The experiment in this manuscript was conducted using the same batch of SERS substrate to avoid uncontrolled variation caused by this variable. Practically, the properties of SERS substrates will change depending on environmental conditions. Thus, further research on SERS substrates must be performed to achieve the goal of real-world application.

## Conclusion

The PCR-SERS protocol, as described within this paper, is an efficient approach for methylation level detection. By decomposing the SERS spectra of PCR products by MLR, the methylation levels of each gene were calculated. In the analysis of plasma samples from 48 NSCLC patients and 51 healthy controls, we found significant methylation profile differences between cancer and control groups at the p16, MGMT, and RASSF1 genes. Prediction accuracy of 88% was achieved using CART analysis based on the methylation levels and states of the three genes, respectively. Fisher's exact test showed that p16 methylation was more frequent in heavy smokers, and was also more frequent in squamous cell and large cell lung cancers than in adenocarcinoma. RASSF1 methylation was found to be more frequent in later stages of NSCLC. Overall, the PCR-SERS method presented here showed itself an efficient methylation level detection method and can be used in clinical plasma gene analysis.

## Supplementary Material

Supplementary figures and tables.

## Figures and Tables

**Table 1:** Sequences of reference dsCG and dsAT strands.

Double-strand	Sequences
dsCG	CCG CGC CGC GCG CGC GGC GCGG
dsAT	AAT ATA ATA TAT ATA TTA TATT

**Table 2:** Clinical characteristics of 48 NSCLC patients and 51 controls.

Clinical features		NSCLC (n=48)	Controls (n=51)
Gender	Female	21 (44%)	27 (53%)
	Male	27 (56%)	24 (47%)
Age	1-40	6 (12%)	6 (12%)
	41-60	7 (15%)	10 (20%)
	61-100	35 (73%)	35 (69%)
Smoking status (pack-years)	0	13 (27%)	22 (43%)
	1-20	13 (27%)	10 (20%)
	21-100	22 (46%)	19 (37%)
TNM stage	I-II	15 (31%)	NA
	III-IV	33 (69%)	NA
Types	Adeno	18 (38%)	NA
	Large cell	6 (12%)	NA
	Squamous	24 (50%)	NA

**Table 3:** Primer sequences used in the PCR for genes p16, MGMT, and RASSF1.

Genes	Primers	lengths (bp)	CpGs	References
p16	F 5'-CGGAGGAAGAAAGAGGAGGGGT-3' R 5'-CGCTACCTACTCTCCCCCTCT-3'	93	7	61
MGMT	F 5'-GGATATGTTGGGATAGTT-3' R 5'-CCCAAACACTCACCAAAT-3'	98	12	62
RASSF1	F 5'-AGTTTGGATTTTGGGGGAGG-3' R 5'-CAACTCAATAAACTCAAACTCCCC-3'	136	12	63

**Table 4:** Specificity, sensitivity, and accuracy for p16, MGMT, and RASSF1 by ROC analysis using Youden's J statistic.

Genes	Specificity	Sensitivity	Accuracy
p16	63%	92%	78%
MGMT	33%	94%	65%
RASSF1	69%	92%	80%

**Table 5:** Results of Fisher's exact test.

Clinical features	p values		
	p16	MGMT	RASSF1
Gender	0.29	0.97	0.49
	0.14	0.76	0.37
Age	0.99	0.27	0.71
	0.90	0.28	0.40
Smoking status	**0.04 (<0.05)**	0.23	0.94
	0.08	0.26	1.00
Cancer stages	0.54	0.73	0.13
	0.35	1.00	**0.03 (<0.05)**
Cancer types	**0.03 (<0.05)**	0.52	0.86
	0.92	0.44	1.00

**Table 6:** Peak assignments.

Wavenumber (cm-1)	Base pairs	Assignments	References
644	CG	G ring str.	28
684	AT	A	64
734	AT	A ring br.	28
788	Both	C ring br., T ring br.	26
1024	Both	2'-deoxyribose, phosphate	65
1098	Both	phosphate str.	28
1188	Both	C ring str., T ring str.	28
1240	Both	C ring str., A ring str.	24, 66
1270	Both	C, A	65
1336	AT	A	66
1354	CG	G	67
1378	Both	G, A, T	65
1484	Both	C, A, T	68
1550	CG	C, G	65
1575	AT	A ring str.	68
1636	Both	carboxyl vibration	28

## Abbreviations

5caC: 5-carboxylcytosine
5fC: 5-formylcytosine
5hmC: 5-hydroxymethylcytosine
5-mC: 5-methylcytosine
CART: classification and regression trees
COBRA: combined bisulfite restriction analysis
DHPLC: denaturing high-performance liquid chromatography
HRM: high resolution melting
LCR: ligase chain reaction
MLR: multiple linear regression
MSP: methylation-specific polymerase chain reaction
MS-SnuPE: methylation-sensitive single nucleotide primer extension
NSCLC: non-small-cell lung cancer
PCR: polymerase chain reaction
ROC: receiver operating characteristic
SERS: surface-enhanced Raman spectroscopy
TET: ten-eleven translocation
