# Supplementary material for: Polymerase chain reaction - surface-enhanced Raman spectroscopy (PCR-SERS) method for gene methylation level detection in plasma
Source: Theranostics. 2020 Jan 1;10(2):898–909. doi: 10.7150/thno.30204 (PMC6929977; doi:10.7150/thno.30204)
Supplement: Supplementary file 1 — Supplementary figures and tables. [file thnov10p0898s1.pdf]

Supplementary Information

Polymerase chain reaction – surface-enhanced Raman spectroscopy (PCR-SERS) method for gene methylation level detection in plasma

Xiaozhou Li[1,2]*[†], Tianyue Yang[1,2†], Caesar Siqi Li[3†], Youtao Song[2]*, Deli Wang[1], Lili Jin[4], Hong Lou[4], Wei Li[5]*

[1]School of Science, Shenyang Ligong University, Shenyang 110159, China

[2]College of Environmental Sciences, Liaoning University, Shenyang 110036, China

[3]College of Medicine, Northeast Ohio Medical University, Rootstown 44272, USA

[4]School of Life Science, Liaoning University, Shenyang 110036, China

[5]School of Electronic Science and Engineering, University of Electronic Science and Technology of China, Chengdu 611731, China

*E-mail: lixiaozhou@lnu.edu.cn (X Li), ysong@lnu.edu.cn (Y Song), Weili@Uestc.edu.cn (W Li)

[†]Contributed equally.

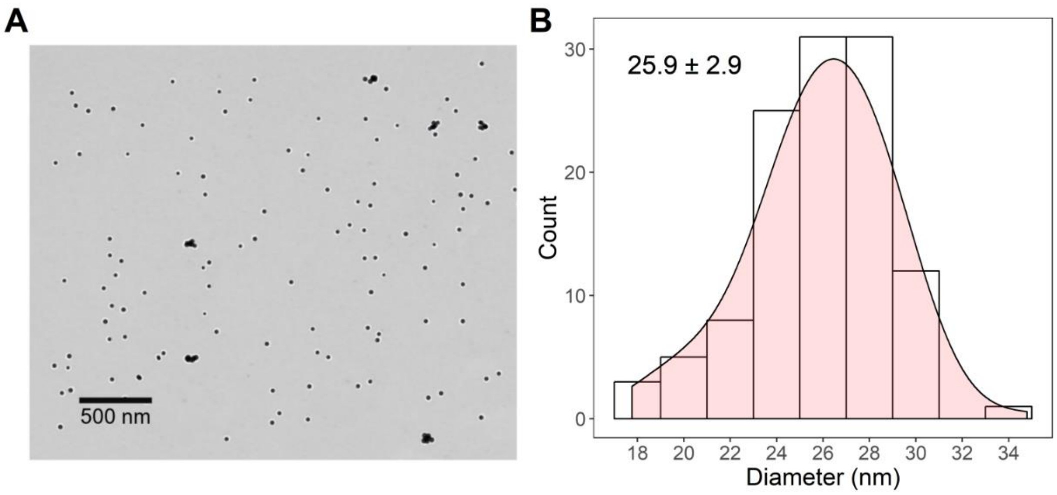

Figure S1. (A) TEM image of silver nanoparticles. (B) Particle size distribution plot.

Table S1. Calculated methylation levels by MLR.

DNA mixtures		MLR coefficients		Actual methylation level	Calculated methylation level	Act-Cal
		dsCG	dsAT			
p16	Mix1	0.555	0.457	0%	0%	0%
	Mix2	0.559	0.457	1%	2%	-1%
	Mix3	0.564	0.455	5%	7%	-2%
	Mix4	0.574	0.438	25%	25%	0%
	Mix5	0.594	0.418	50%	52%	-2%
	Mix6	0.619	0.407	75%	73%	2%
	Mix7	0.637	0.381	100%	103%	-3%
MGMT	Mix1	0.35	0.668	0%	-3%	3%
	Mix2	0.354	0.666	1%	0%	1%
	Mix3	0.356	0.656	5%	4%	1%
	Mix4	0.381	0.637	25%	22%	3%
	Mix5	0.416	0.602	50%	51%	-1%
	Mix6	0.447	0.576	75%	73%	2%
	Mix7	0.479	0.548	100%	98%	2%
RASSF1	Mix1	0.375	0.623	0%	1%	-1%
	Mix2	0.377	0.629	1%	0%	1%
	Mix3	0.383	0.625	5%	6%	0%
	Mix4	0.398	0.603	25%	26%	-1%
	Mix5	0.424	0.586	50%	51%	-1%
	Mix6	0.445	0.564	75%	74%	1%
	Mix7	0.469	0.54	100%	102%	-2%

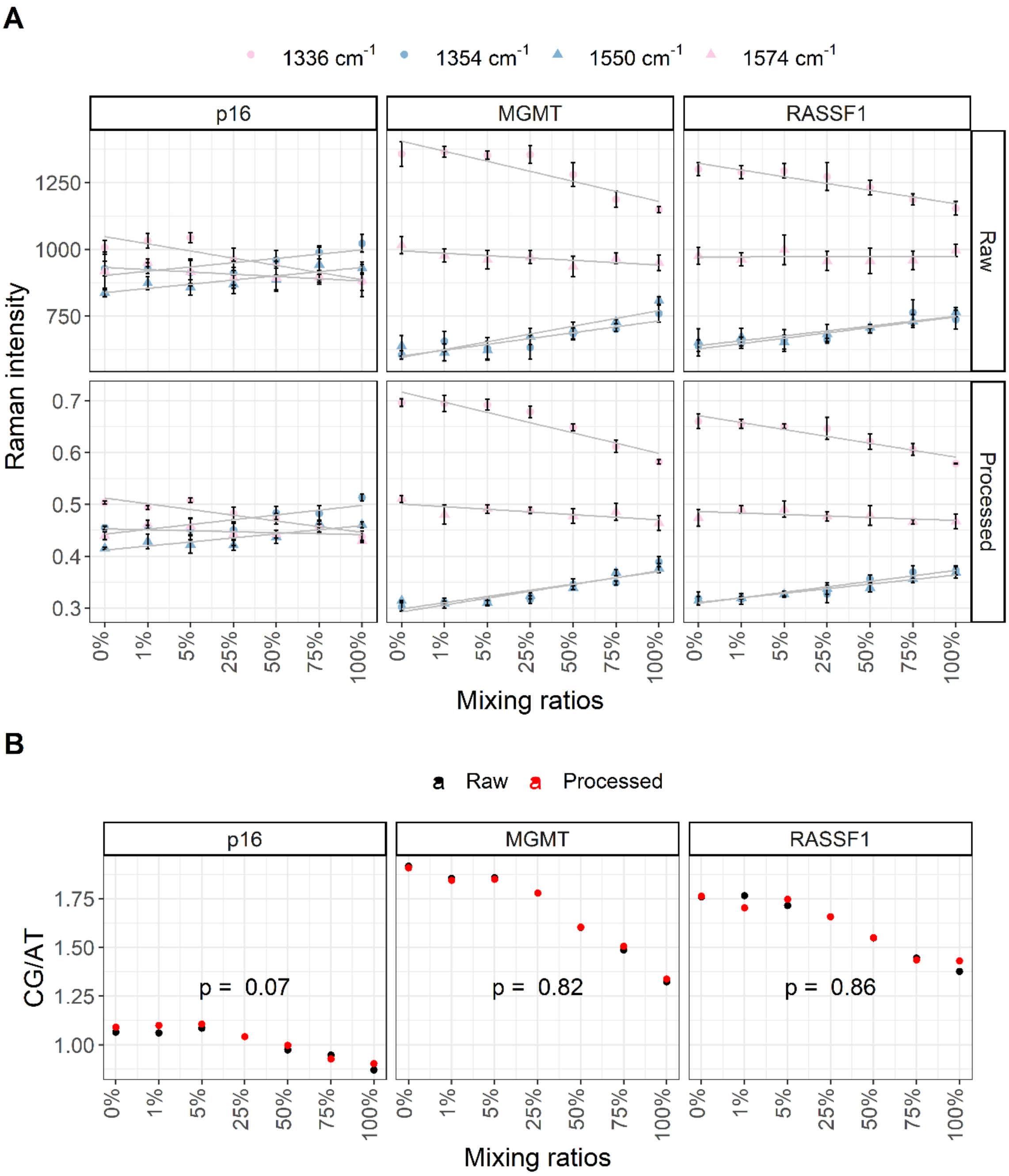

Figure S2. Comparison between raw spectra and processed spectra. (A) Peak heights and changing trends of 1336, 1354, 1550, and 1574 cm^{-1} of raw and processed spectra. (B) Averaged peak heights of CG/AT of the genes p16, MGMT, and RASSF1 of raw (black) and processed (red) spectra. p values of the paired T-test of the three genes were all larger than 0.05 (p=0.07, 0.82, and 0.86) which shows no significant difference existed between the raw and processed spectra.

Table S2. Comparison between methylation detection methods.

Methods	Information provided	Pretreatment	Amplification	Separation	Detection	Refs
**Polymerase chain reaction – surface-enhanced Raman spectroscopy (PCR-SERS in this paper)**<br>• Pro: Rapid, low-cost<br>• Cons: Reproducibility of SERS substrate	Quantitative<br>Methylation levels (percentages) between primers	Bisulfite<br>• Function: convert unmethylated cytosines (C) to uracil (U)	PCR<br>• Run time: 2-3 hours<br>• Primers: Non-methylation specific primers (do not contain CpG sites)	SERS<br>• Run time: About 1 minute<br>• Principle: By comparing peak heights of C and T to deduce the C/T ratio and subsequently deduce the methylation percentages (levels)		
Methylation-sensitive high resolution melting (MS-HRM)<br>• Pros: In-tube detection, rapid, low cost<br>• Cons: Design of primers		Bisulfite	PCR<br>• Run time: 2-3 hours<br>• Primers: Non-methylation specific primers	HRM<br>• Run time: About 3 minutes<br>• Principle: Detecting methylation by measuring intercalating fluorescent dyes liberated under heating. The degree of methylation can be evaluated by the shape of the melting curve		[1]
Methylation-specific polymerase chain reaction (MSP)<br>• Pro: Can be incorporated into other biological techniques<br>• Cons: Design of primers	Qualitative<br>Methylation existence states of predetermined CpG sites	Bisulfite	MSP/nested MSP<br>• Run time: 1-2/5-7 hours<br>• Primers: Methylation-specific primers (containing CpG sites)	Gel electrophoresis<br>• Run time: 1-2 hours<br>• Principle: Separate DNA fragments with or without methylated CpGs by size and charge		[2, 3]
Methylation-sensitive denaturing high-performance liquid chromatography (MS-DHPLC)<br>• Pro: High sensitivity, low cost<br>• Cons: Temperature selection		Bisulfite	PCR<br>• Run time: 2-3 hours<br>• Primers: Non-methylation specific primers	DHPLC<br>• Run time: 5-10 minutes<br>• Principle: First use denaturation-renaturation to create hetero- and homoduplexes from PCR products containing methylated CpGs. Then use differential retention of homo- and heteroduplexes to differentiate DNA fragments with or without methylated CpGs		[4]
Methylation-sensitive single nucleotide primer extension (MS-SnuPE)<br>• Pros: Sensitive, quantitative<br>• Cons: PCR bias and analyses in CpG-rich regions can be a problem	Quantitative<br>Methylation quantity of single predetermined CpG site	Bisulfite	PCR<br>• Run time: 2-3 hours<br>• Primers: Non-methylation specific primers	SnuPE<br>• Run time: 2-3 hours<br>• Principle: The primer is allowed to extend one base pair (C or T) in the presence of DNA polymerase terminators, and the ratio of C/T is determined for the evaluation of methylation	Fluorescence/Pyrosequencing/matrix-assisted laser desorption ionization/time-of-flight (MALDI-TOF) mass spectrometry/Ion pair reverse-phase high-performance liquid chromatography (IP-RP-HPLC)<br>• Principle: Use fluorescence, pyrosequencing, mass spectroscopy or HPLC to measure the C:T ratio	[5, 6]
Combined bisulfite Restriction analysis (COBRA)<br>• Pros: Fast, high-throughput, and economic<br>• Cons: Limited to existing restriction sites	Qualitative or quantitative<br>Methylation of predetermined CpG sites	Bisulfite	PCR<br>• Run time: 2-3 hours.<br>• Primers: Non-methylation specific primers	Restriction digest<br>• Run time: 30–40 hours<br>• Principle: To cleave part of PCR products that having methylated CpG sites using restriction enzymes	Gel electrophoresis/Pyrosequencing<br>• Principle: Separate digested DNA fragments using different length, or do pyrosequencing on the digested products	[7, 8]
MethyLight<br>• Pro: Sensitive, high-throughput<br>• Cons: High cost	Quantitative<br>Methylation quantity of predetermined CpG sites	Bisulfite	Real-time PCR (RT-PCR)<br>• Run time: 2-3 hours<br>• Primers: One of or both primers and probes are methylation-specific<br>• Principle: Methylation quantification is achieved by the ratio between methylated reactions and control reactions.			[9, 10]
Bisulfite-Sequencing<br>• Pros: Massively parallel detection.<br>• Cons: Challenging, high cost	Quantitatve<br>Methylation of all CpG sites	Bisulfite	PCR/nested PCR<br>• Run time: 1-2/5-7 hours<br>• Primers: Non-methylation specific sequencing primers	Direct sequencing<br>• Run time: 4-60 hours<br>• Principle: By comparing the sequencing read from methylated reactions and control reactions		[11, 12]

References

1. Wojdacz T, Dobrovic A. Methylation-sensitive high resolution melting (MS-HRM). Nucleic Acids Res. 2007; 35: e41.

2. Licchesi J, Herman J. Methylation-specific PCR. Methods Mol Biol. 2009; 507: 305–323.

3. Herman J, Graff J, Myohanen S, Nelkin B, Baylin S. Methylation-specific PCR. Proc Natl Acad Sci U S A. 1996; 93: 9821–9826.

4. Xiao W, Oefner P. Denaturing high-performance liquid chromatography. Hum Mutat. 2001; 17: 439–474.

5. Nikolausz M, Chatzinotas A, Táncsics A, Imfeld G, Kästner M. The single-nucleotide primer extension (SNuPE) method for the multiplex detection of various DNA sequences. Biochem Soc Trans. 2009; 37: 454–459.

6. Hou P, Ji M, Chen Z, Lu Z. A profile of current methods for DNA methylation analysis. Curr Anal Chem. 2006; 2: 309–322.

7. Xiong Z, Laird P. COBRA: a sensitive and quantitative DNA methylation assay. Nucleic Acids Res. 1997; 25: 2532–2534.

8. Fraga M, Esteller M. DNA methylation: a profile of methods and applications. Biotechniques. 2002; 33: 632, 634, 636-49.

9. Eads C, Danenberg K, Kawakami K, Saltz L, Blake C, Shibata D, et al. MethyLight: a high-throughput assay to measure DNA methylation. Nucleic Acids Res. 2000; 28: E32.

10. Trinh B, Long T, Laird P. DNA methylation analysis by MethyLight technology. Methods. 2001; 25: 456–462.

11. Frommer M, McDonald L, Millar D, Collis C, Watt F, Grigg G, et al. A genomic sequencing protocol that yields a positive display of 5-methylcytosine residues in individual DNA strands. Proc Natl Acad Sci U S A. 1992; 89: 1827–1831.

12. Li P, Demirci F, Mahalingam G, Demirci C, Nakano M, Meyers B. An integrated workflow for DNA methylation analysis. J Genet Genomics. 2013; 40: 249–260.
